# Supplementary figures and images for: Pipeline to evaluate YAP-TEAD inhibitors indicates TEAD inhibition represses NF2-mutant mesothelioma
Source: Life Sci Alliance. 2025 Jul 31;8(10):e202503241. doi: 10.26508/lsa.202503241 (PMC12314556; doi:10.26508/lsa.202503241)

## Figure 1 – Source Data

### YAP Phosphorylation - MeT-5A

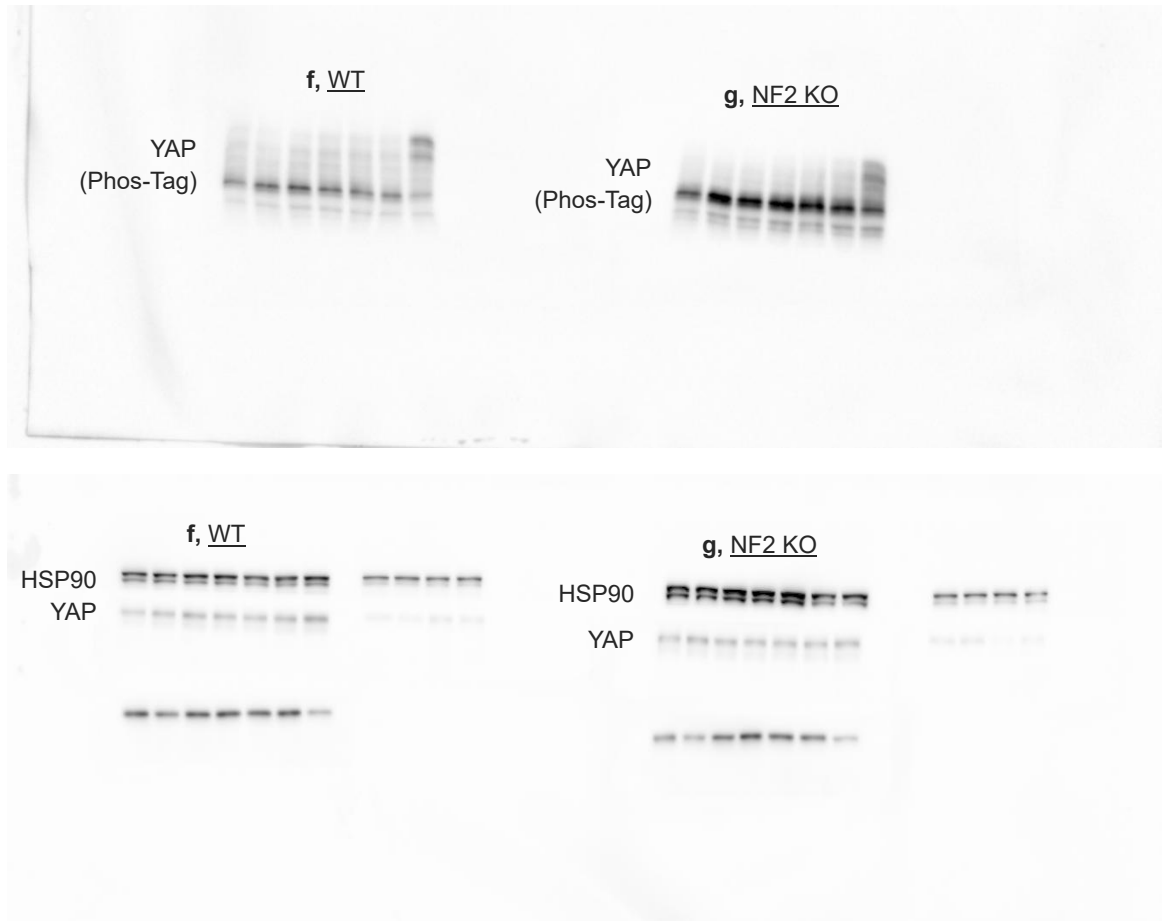

Supplement: Supplementary file 1 [file LSA-2025-03241_SdataF1.zip › Fig1/WB-Source.pdf]

## Supplementary Figure S1 – Source Data

### YAP Phosphorylation – MeT-5A

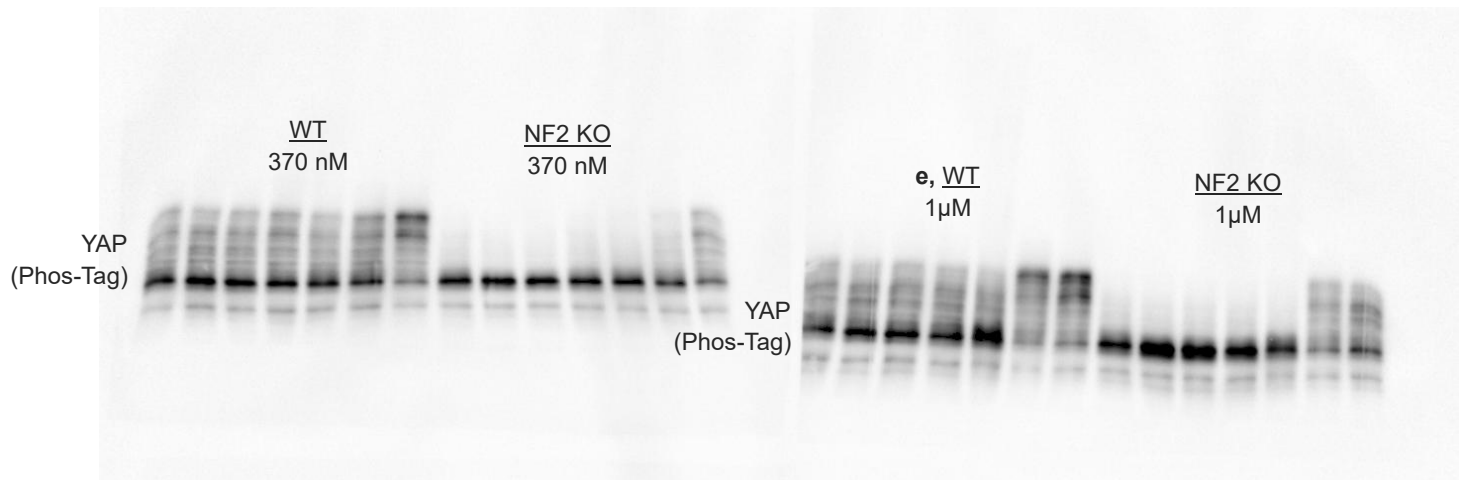

## YAP Phosphorylation – HEK293A

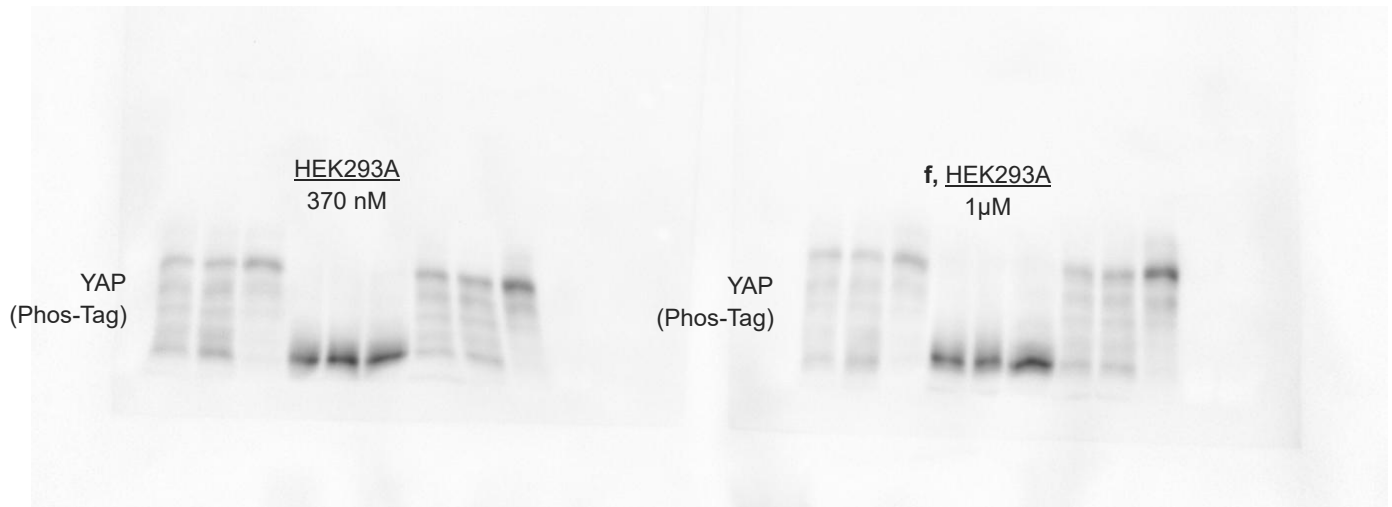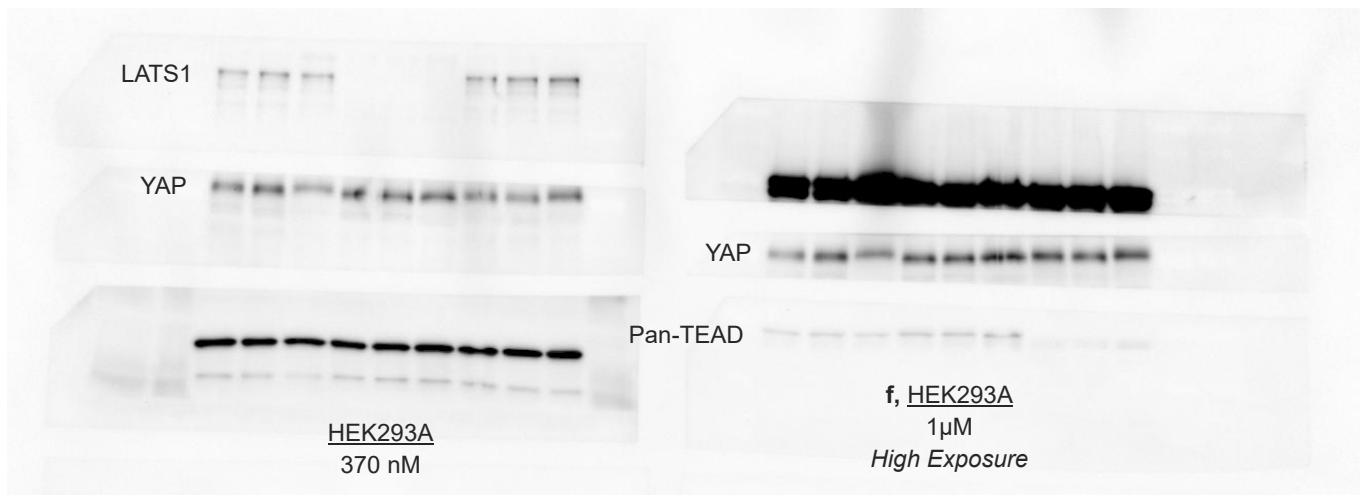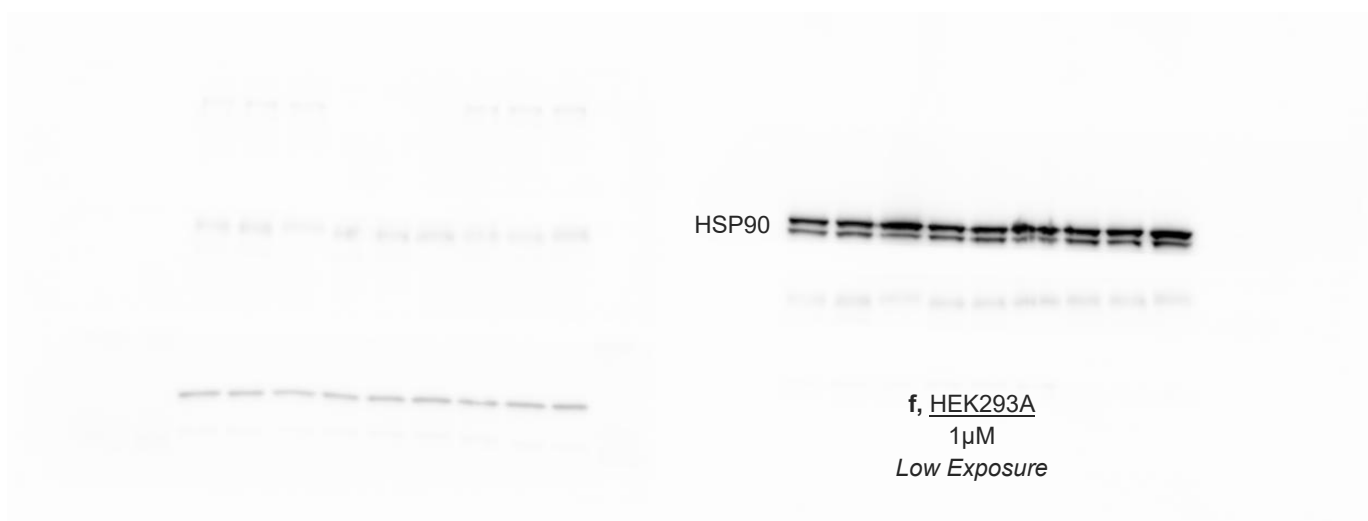

Supplement: Supplementary file 2 [file LSA-2025-03241_SdataFS1.1.zip › FigS1/WB-Source.pdf]
