# Supplementary material for: Pipeline to evaluate YAP-TEAD inhibitors indicates TEAD inhibition represses NF2-mutant mesothelioma
Source: Life Sci Alliance. 2025 Jul 31;8(10):e202503241. doi: 10.26508/lsa.202503241 (PMC12314556; doi:10.26508/lsa.202503241)

Figure 3 – Source Data

YAP-5SA Expression – Validation & NF2 dKO Validation

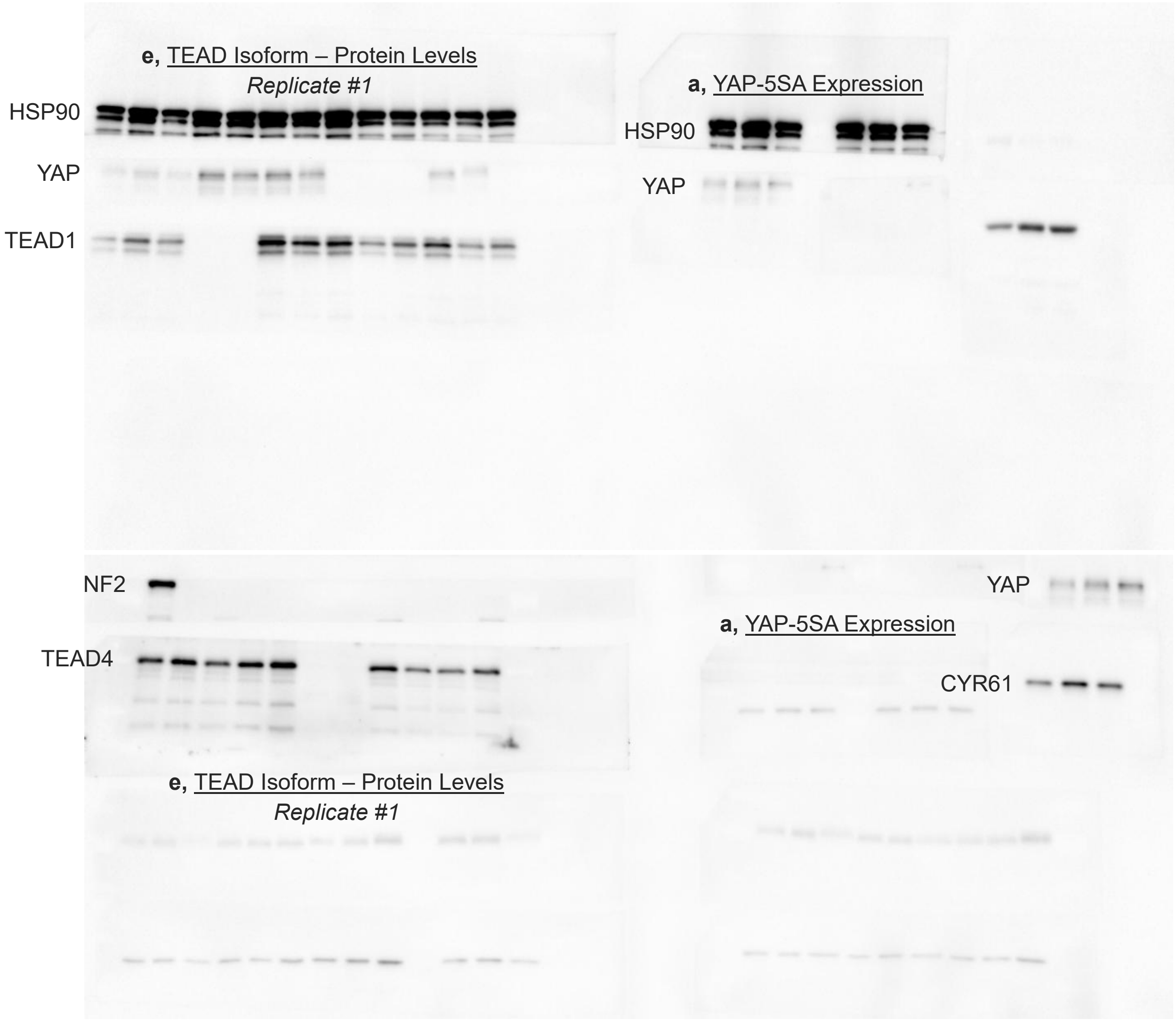

TEAD Isoform – Protein Levels

e, TEAD Isoform – Protein Levels
Replicate #2

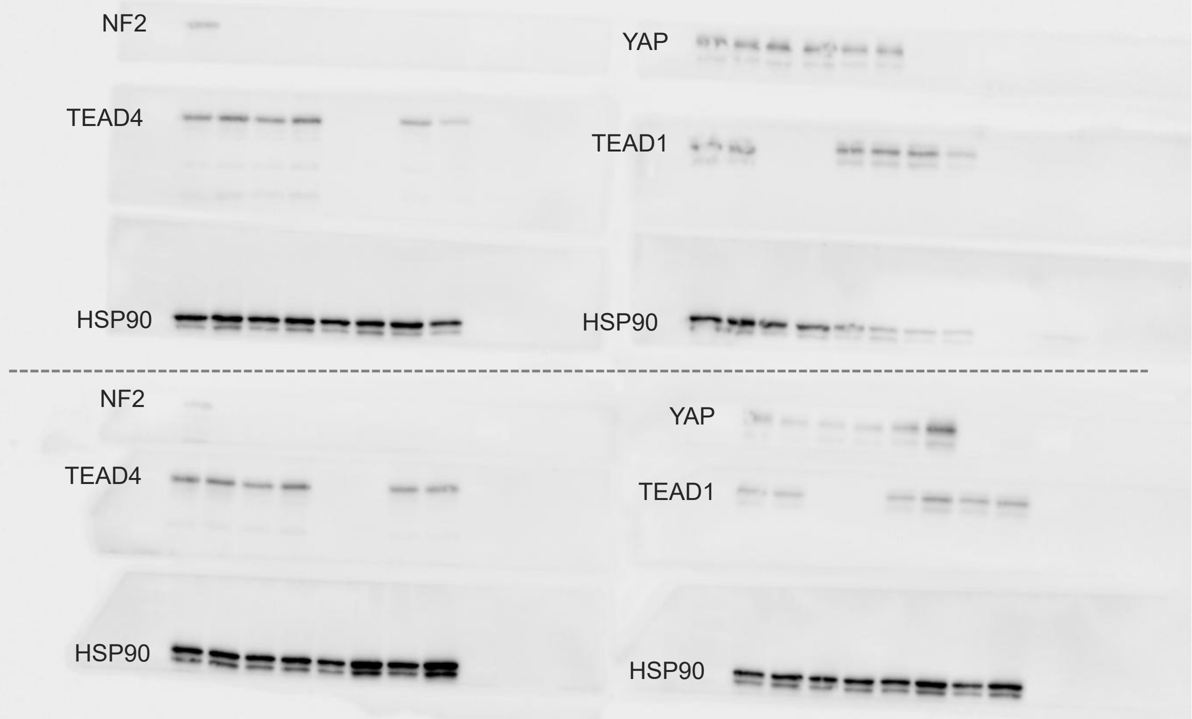

d-e, NF2 dKO Validation
Replicate #3 (Low Exposure)

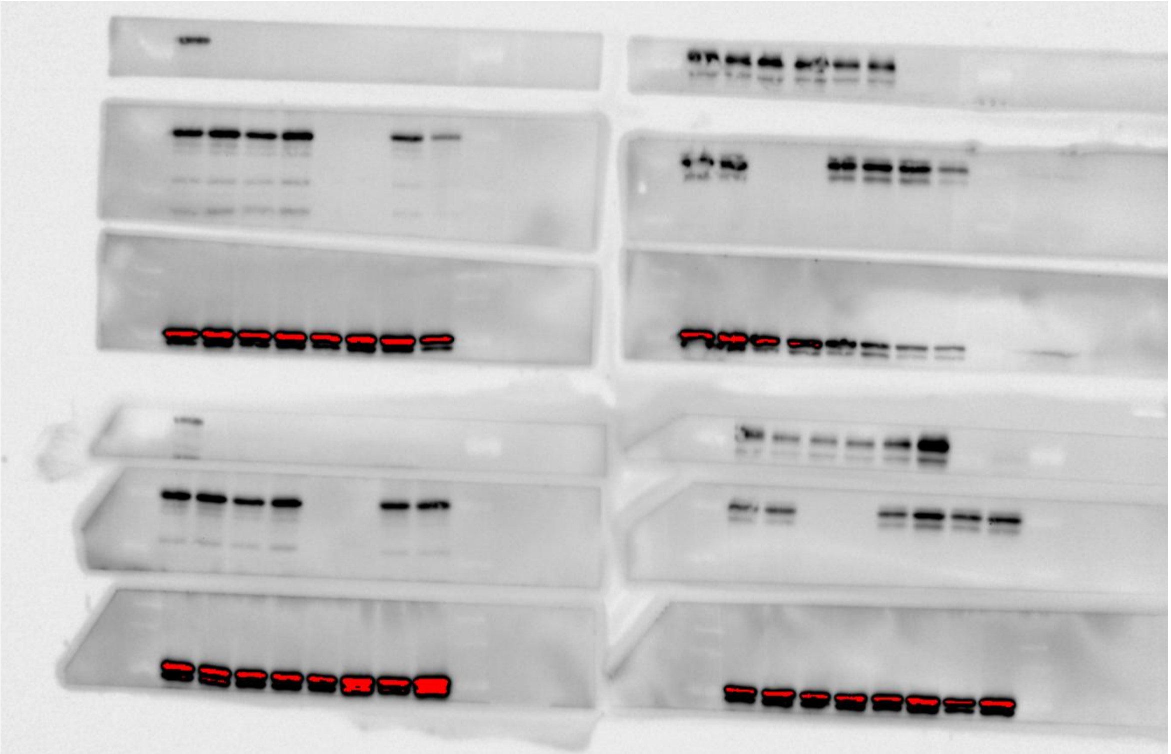

d-e, NF2 dKO Validation
Replicate #3 (High Exposure)

e, TEAD Isoform – Protein Levels
Replicate #4

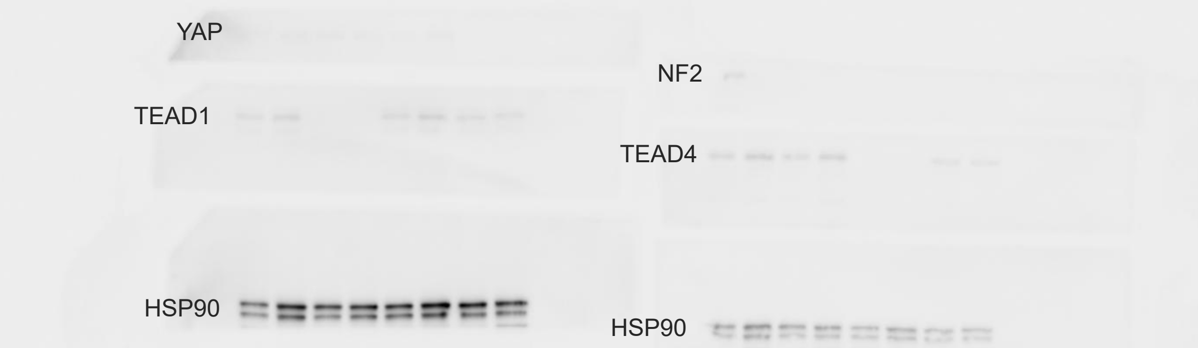

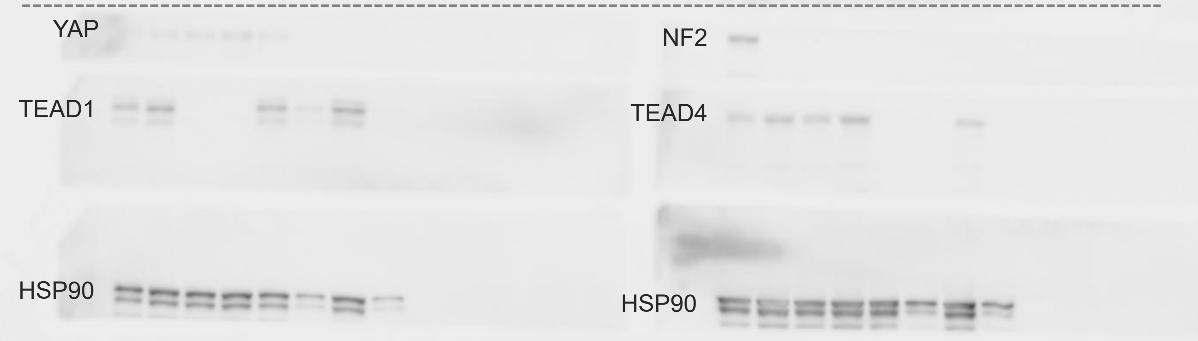

e, TEAD Isoform – Protein Levels
Replicate #5

e, TEAD Isoform – Protein Levels
Replicate #6

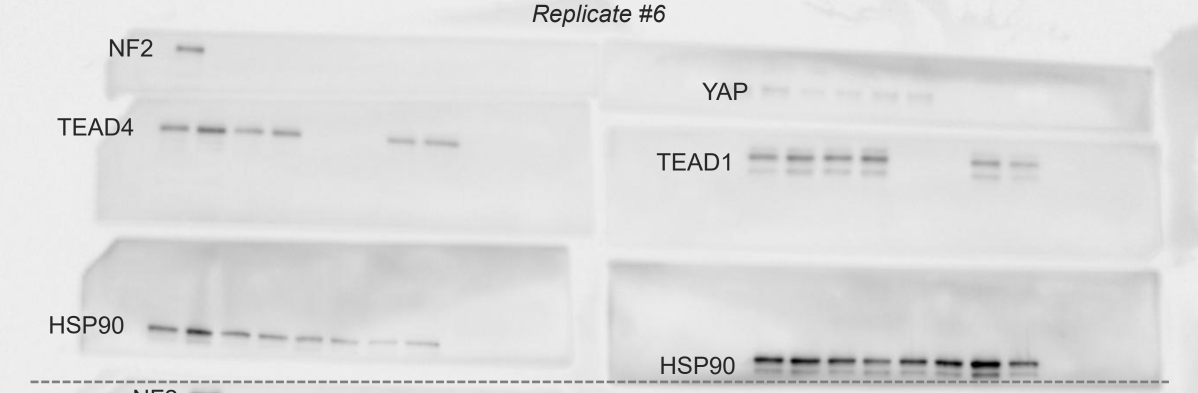

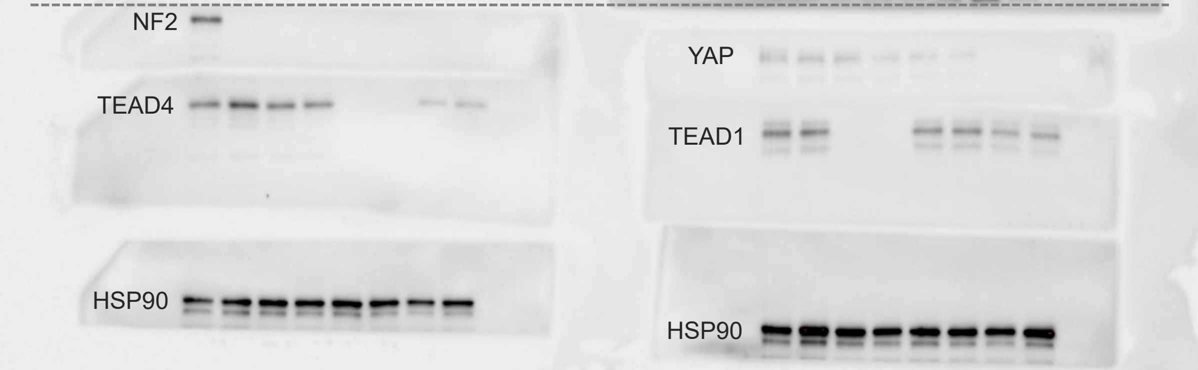

e, TEAD Isoform – Protein Levels
Replicate #7

e, TEAD Isoform – Protein Levels

Replicate #8

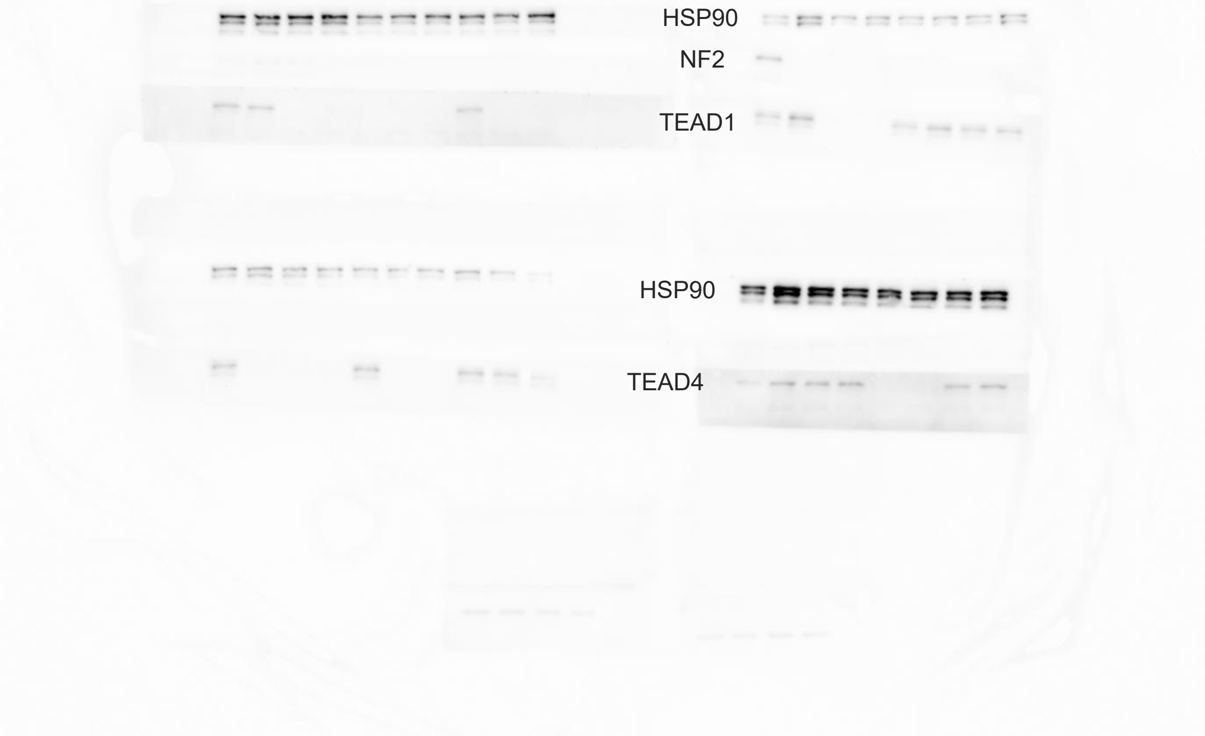

Supplement: Supplementary file 5 [file LSA-2025-03241_SdataF3.zip › Fig3/WB-Source.pdf]
